# Beneficial Effects of Human Anti-Interleukin-15 Antibody in Gluten-Sensitive Rhesus Macaques with Celiac Disease

**DOI:** 10.3389/fimmu.2018.01603

**Published:** 2018-07-11

**Authors:** Karol Sestak, Jason P. Dufour, David X. Liu, Namita Rout, Xavier Alvarez, James Blanchard, Anne Faldas, David J. Laine, Adam W. Clarke, Anthony G. Doyle

**Affiliations:** ^1^Division of Microbiology, Tulane National Primate Research Center, Covington, LA, United States; ^2^PreCliniTria LLC, Mandeville, LA, United States; ^3^Division of Veterinary Medicine, Tulane National Primate Research Center, Covington, LA, United States; ^4^Division of Clinical Research, Integrated Research Facility, National Institute of Allergy and Infectious Disease, Frederick, MD, United States; ^5^Division of Immunology, Tulane National Primate Research Center, Covington, LA, United States; ^6^Division of Comparative Pathology, Tulane National Primate Research Center, Covington, LA, United States; ^7^Teva Pharmaceuticals, R&D, Biologics, Lead Antibody Discovery, Sydney, NSW, Australia

**Keywords:** gluten, celiac disease, interleukin-15, antibody therapy, small intestine

## Abstract

Overexpression of interleukin-15 (IL-15) is linked with immunopathology of several autoimmune disorders including celiac disease. Here, we utilized an anti-human IL-15 antibody 04H04 (anti-IL-15) to reverse immunopathogenesis of celiac disease. Anti-IL-15 was administered to six gluten-sensitive rhesus macaques with celiac disease characteristics including gluten-sensitive enteropathy (GSE), and the following celiac-related metrics were evaluated: morphology (villous height/crypt depth ratio) of small intestine, counts of intestinal intraepithelial lymphocytes, IFN-γ-producing CD8+ and CD4+ T cells, plasma levels of anti-gliadin and anti-intestinal tissue transglutaminase IgG antibodies, as well as peripheral effector memory (CD3+CD28−CD95+) T cells. Anti-IL-15 treatment reversed the clinically relevant disease endpoints, intraepithelial lymphocyte counts, and villous height/crypt depth ratios within jejunal biopsies to normal levels (*P* < 0.001). Additionally, intestinal CD8+ and CD4+ T cell IFN-γ production was reduced (*P* < 0.05). Extra-intestinally, anti-IL-15 treatment reduced peripheral NK cell counts (*P* < 0.001), but otherwise, non-NK peripheral lymphocytes including effector memory T cells and serum blood chemistry were unaffected. Overall, providing the beneficial disease-modulatory and immunomodulatory effects observed, anti-IL-15 treatment might be considered as a novel therapy to normalize intestinal lymphocyte function in celiac disease patients with GSE.

## Introduction

Celiac and non-celiac gluten sensitivities are worldwide on the rise, and in the U.S. alone responsible for over 20 million cases requiring therapeutic care (1). In clinical studies, it was shown that populations of the small intestinal intraepithelial lymphocytes (IELs), namely the cytotoxic T lymphocytes (CTL), NK, and NKT cells play important roles in progression toward celiac enteropathy, i.e., destruction of enterocytes followed by villous atrophy (2, 3). During this process, CTL in particular, are reprogrammed, becoming T cell receptor (TCR)-independent cells that exhibit characteristics of the NK (NKT) cells (3). These cells contribute to the destruction of transglutaminase (TG2) autoantigen-secreting MICA+ enterocytes *via* NKG2D receptor, independent of MHC I (self) recognition (4, 5). Such events drive the celiac-specific intestinal pathology, also known as gluten-sensitive enteropathy (GSE) that varies in severity in different individuals and stages of the disease (6).

In murine models, it was established that IL-15 is responsible for the development, maintenance, and expansion of CTL, NK, and NKT cells (7–9). The overexpression of IL-15 in the small intestine of a gluten-sensitive patient is considered one of the key hallmarks of celiac disease (10). There is a consensus that IL-15, *via* promotion of intestinal NK and NKT cells, contributes to celiac disease pathogenesis, namely GSE. In IL-15-overexpressing transgenic mice enteropathy models, IL-15 blockade with anti-IL-15 antibody was shown to reverse intestinal damage (11). However, transgenic mouse models of celiac disease including those with major histocompatibility complex class II alleles do not reproduce unique and complex aspects of the human disease. Therefore, to examine the involvement of IL-15 in a model that is more representative of human celiac disease, we used the primate (rhesus macaque) model of GSE. Our group previously reported IL-15 small intestinal overexpression as well as IL-17/22 dis-regulation and MHC II genetic predisposition in rhesus macaques with celiac disease characteristics (12, 13). In celiac macaques, IL-15 signal was demonstrated within and beneath the small intestinal epithelium in lamina propria. A relationship was also suggested between IL-15 expression and altered gut microflora, which in turn can negatively impact gut function (14, 15).

While experimental anti-IL-15 antibody administration into rhesus and cynomolgus macaques has been described to impact NK and T cell homeostasis (16, 17), these parameters have not yet been interrogated in a macaque host in the context of GSE. Due to close similarities in pathogenesis and immunology with human celiac disease, the rhesus macaque model (18–20) was used to evaluate the efficacy of anti-IL-15 treatment in this study. Quantitative measurements of intestinal IELs, T lymphocytes producing IFN-γ, plasma anti-gliadin and anti-TG2 antibodies, peripheral NK and T cells, together with evaluation of small intestinal tissue architecture were all used as metrics. We show that anti-IL-15 treatment targets important lymphoid cells such as small intestinal IELs and inflammatory CD3+ T lymphocytes that are known to contribute to pathogenesis of celiac disease, and as such, anti-IL-15 might be considered as a candidate for novel supportive therapy, especially in patients suffering with a severe form of disease where traditional (gluten-free diet) approach is not sufficient.

## Materials and Methods

### Rhesus Macaques

Six young (1.2–2.3 years of age) rhesus macaques (*Macaca mulatta*) of Indian origin with human celiac disease characteristics (positive for anti-gliadin antibodies (AGA) and TG2 antibodies), three of them with mild (Type 1) enteropathy characterized by intraepithelial lymphocytosis but not villous atrophy, i.e., Group 1 (LD17, LF68, and LE73) and three animals with moderate (Type 2) enteropathy characterized by intraepithelial lymphocytosis with villi present but shortened, i.e., Group 2 (LE99, LD09, and LI75) were used as treatment subjects. Animals were selected for the study out of 335 candidates during Tulane colony semi-annual health screenings. In addition, three age-matched, AGA antibody-negative, clinically healthy macaques fed conventional monkey chow (gluten-containing diet), were used as controls to establish the immunological baselines. Animals were selected irrespective of sex, kept under the bio-security level 2 at TNPRC, and were seronegative and free of viral, bacterial or parasitic pathogens including the simian retrovirus type D, simian T lymphotropic virus type 1, simian immunodeficiency virus (SIV) and herpes B virus. Tuberculin skin tests were negative for each individual. Prior to their study assignment, all macaques were fed regular, gluten-containing monkey chow (Purina) as described in detail elsewhere (12, 20).

### Diets, Anti-IL-15 Treatment, and Samples Collected

**Figure 1:**
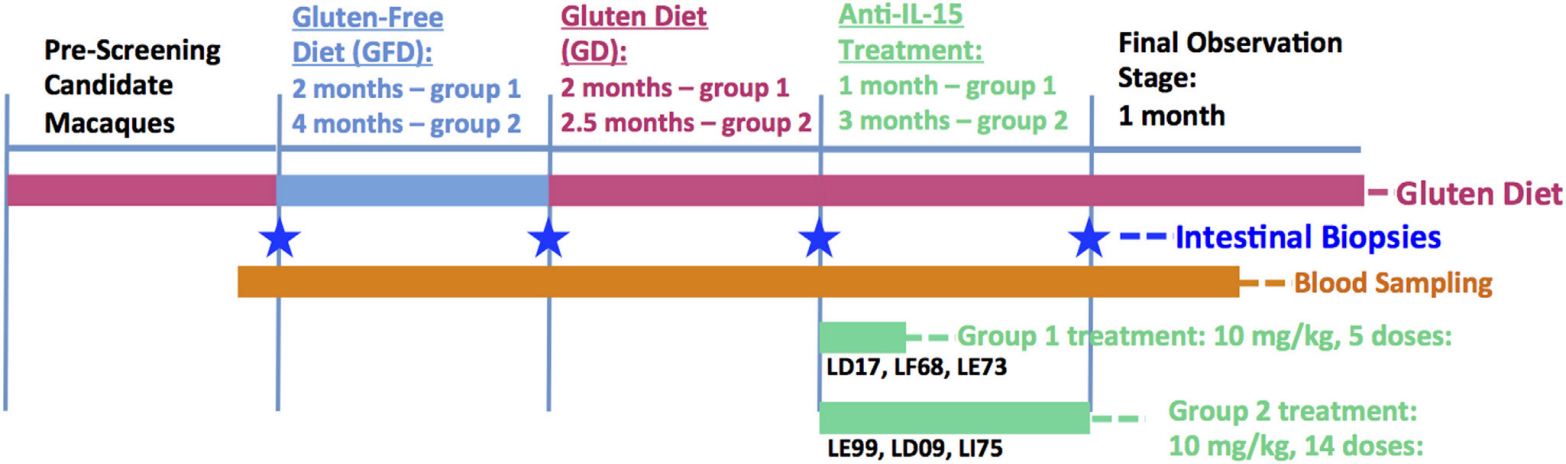
Study design. Lengths of each diet and treatment period were adjusted for group 1 and group 2 celiac macaques based on remission of anti-gliadin antibodies and/or transglutaminase antibodies and gluten-sensitive enteropathy. In general, due to more severe disease (enteropathy) in group 2 macaques, both GFD and anti-IL-15 treatment periods were extended in this group compared with group 1.

The gluten-free (GFD) and gluten-containing (GD) diets were administered to all six macaques to induce the stages of immunological remission and relapse, respectively, characterized by anti-gliadin/-TG2 positive and negative plasma antibody responses as described (12, 13, 20). After inducing immunological relapse, human anti-IL-15 antibody 04H04 (Teva Pharmaceuticals) was intravenously (i.v.) administered weekly in a dose of 10 mg/kg (BW) to three macaques for 28 days (Group 1) and three macaques for 90 days (Group 2) while macaques were still fed GD (Figure 1). All six macaques started experiment as one “celiac-like” group that was later subdivided into two groups based on enteropathy. In general, due to more severe enteropathy in group 2 macaques, both GFD and anti-IL-15 treatment periods were extended in this group.

Samples collected prior, during and post anti-IL-15 treatment included bi-weekly blood (5 ml EDTA) for serum chemistries and CBC counts. One milliliter of whole blood without anti-coagulant was collected prior to each of the i.v. administrations to obtain serum for PK analyses. PK analysis was performed using the enzyme-linked immunoassay method described by Yang and colleagues (21). Small-intestinal biopsies were taken at four defined time points, i.e., at the end of each diet/treatment period (GD, GFD, second GD/challenge, and anti-IL-15 treatment) in accord with sampling procedures described previously (12).

### Antibody Assays, Histopathological and Morphometric Evaluation

The evaluation of anti-gliadin and TG2 plasma (IgG) antibodies, GSE including morphometric evaluation of small intestinal villous heights vs. crypt depths, i.e., V/C ratios, was done according to previously established protocols (12, 13, 20, 22, 23), consistent with considerations regarding morphometric analysis of celiac biopsies (24). Briefly, the villus height vs. crypt depth ratios were obtained from linear measurements of five villus heights per animal and time point, divided by the corresponding crypt depths. The linear measurement of the villus height was made from the top of the villi to the mouth of the crypt of Lieberkuhn. The crypt depth was measured as the distance from the mouth of the crypt of Lieberkuhn to the upper border of intestinal lamina muscularis. Microscopic evaluations of hematoxilin/eosin-stained small intestinal tissues from study macaques were performed blinded to the sample source (20).

### Confocal Microscopy and Quantitative Evaluation of IELs

Small intestinal biopsies were collected at times corresponding to immunological relapse (GD) and remission (GFD), as well as the beginning and end of the anti-IL-15 treatment period. Biopsies were collected and processed as described (12, 13, 23). Briefly, tissues were embedded in paraffin and 7 µm sections were stained first with unconjugated primary antibodies, including (1) cytokeratin 1, i.e., an epithelial cell marker (CKLMW 8/18, Biocare Medical, Concord, CA, USA), (2) CD3, i.e., a T cell marker (CD215C, Dako, Carpinteria, CA, USA); and To-Pro 3, i.e., a nuclear DNA marker (T-3605, Invitrogen, Carlsbad, CA, USA). Primary antibodies were followed by appropriate secondary, fluorochrome-conjugated antibodies. Confocal microscopy with a Leica TCS SP8 laser-scanning system was used to collect the images as described (12). Image analysis was performed with Volocity software (version 6.3, PerkinElmer, Waltham, MA, USA) to enumerate the CD3+ cells inside the 10 defined (1 mm^2^) squares per each animal’s small intestinal epithelium and lamina propria sample, i.e., IELs and LPLs, respectively.

### Fluorescent-Activated Cell Sorting (FACS)

Evaluation of the impact of anti-IL-15 treatment on presence of rhesus NK and T cells including intestinal cytotoxic T (CD8+), helper T (CD4+) cells producing IFN-γ, and peripheral effector memory T (CD3+CD28−CD95+) cells was done by use of commercial, human-specific, rhesus-cross-reactive antibodies (Table 1). Lymphoid cells were isolated from peripheral blood by use of lymphocyte separation medium following the manufacturer’s protocol (LSM, MP Biomedicals, Solon, OH, USA) and small intestinal tissue biopsies as described (12, 13). Briefly, intestinal biopsy samples (obtained by endoscopy or laparotomy) were processed using the enzymatic (EDTA-collagenase) digestion method: IELs were isolated by EDTA and mechanical agitation, and LPLs by collagenase. Due to relatively small size of endoscopic biopsies (0.2–0.3 cm), isolated IELs and LPLs were always pooled. Following Percoll (Life Sciences, Marlborough, MA, USA) density gradient centrifugation step, IEL contamination in the CD45 + LPL population in the lower layer of density gradient was between 1.0 and 3.0%. Intestinal cell viability was >90%, as determined by trypan blue dye exclusion method.

**Table 1:** List of antibodies used in fluorescent-activated cell sorting.

Antigen	Clone	Label	Source	City, state
CD3	SP34-2	AL 700	BD Pharmingen	San Jose, CA
CD3	CD3-12	Pacific Blue	Bio-Rad	Hercules, CA
CD4	OKT4	BV 510	Bio Legend	San Diego, CA
CD8a	SK1	APC-H7	BD Pharmingen	San Jose, CA
CD16	3G8	AL 488	Bio Legend	San Diego, CA
CD28	CD28.2	PerCP-Cy5.5	BD Pharmingen	San Jose, CA
CD45	D058-1283	BV 711	BD Biosciences	San Jose, CA
CD56	NCAM 16	BV 650	BD Horizon	San Jose, CA
CD95	DX2	BV 711	BD Biosciences	San Jose, CA
HLADR	L243	PerCP-Cy5	Bio Legend	San Diego, CA
NKG2D	1D11	AL 647	Bio Legend	San Diego, CA
IFN-γ	4S.B3	PE-Cy7	BD Pharmingen	San Jose, CA

Isolated lymphoid cells were stained for surface markers the day of sampling and cell suspensions were kept on ice between each step to prevent changes in cell surface expression (13). To detect the intracellular (IFN-γ) cytokine production, cells were prior to first (surface) staining *in vitro* stimulated with 0.1 µM PMA and 0.5 µg/ml ionomycin (Sigma, St. Louis, MO, USA) (13). Cells were then stained with antibodies against surface antigens (Table 1), fixed in 1× BD Stabilizing Fixative (BD, San Jose, CA, USA), permeabilized with 1× BD Cytofix/Cytoperm solution, washed with 1× phosphate saline buffer, stained for intracellular proteins, washed and final-fixed in 1× BD Stabilizing fixative. Samples were stored at 4°C for ≤3 days before the data were acquired by FACSAria flow cytometer (BD). Acquired data were analyzed by Flowjo X software (Flowjo LLC, Ashland, OR, USA). Blood NK cells were defined as CD45+ lymphocytes that were CD3−HLADR−CD8α+NKG2D+ and then further delineated as CD56+/CD16+ cells. Intestinal T cells producing IFN-γ were defined as inflammatory CD3+ T lymphocytes that were either CD45+CD4−CD8+IFN-γ+ or CD45+CD4+CD8−/+IFN-γ+ (12).

### Statistical Analysis

Graphical representation and statistical analysis of the IEL counts, LPL/IEL ratios, V/C ratios, and FACS phenotype frequencies (%) were performed by GraphPad Prism 7.0 software and One-Way ANOVA analysis (GraphPad, La Jolla, CA, USA). Comparisons between the time-points associated with GFD, GD diets and anti-IL-15 treatment were done for AGA and TG2 antibody measurements by Mann–Whitney *U* test. Values of *P* < 0.05 were considered significant.

## Results

### Anti-IL-15 Treatment Improves Small Intestinal Tissue Architecture in Macaques with GSE

**Figure 2:**
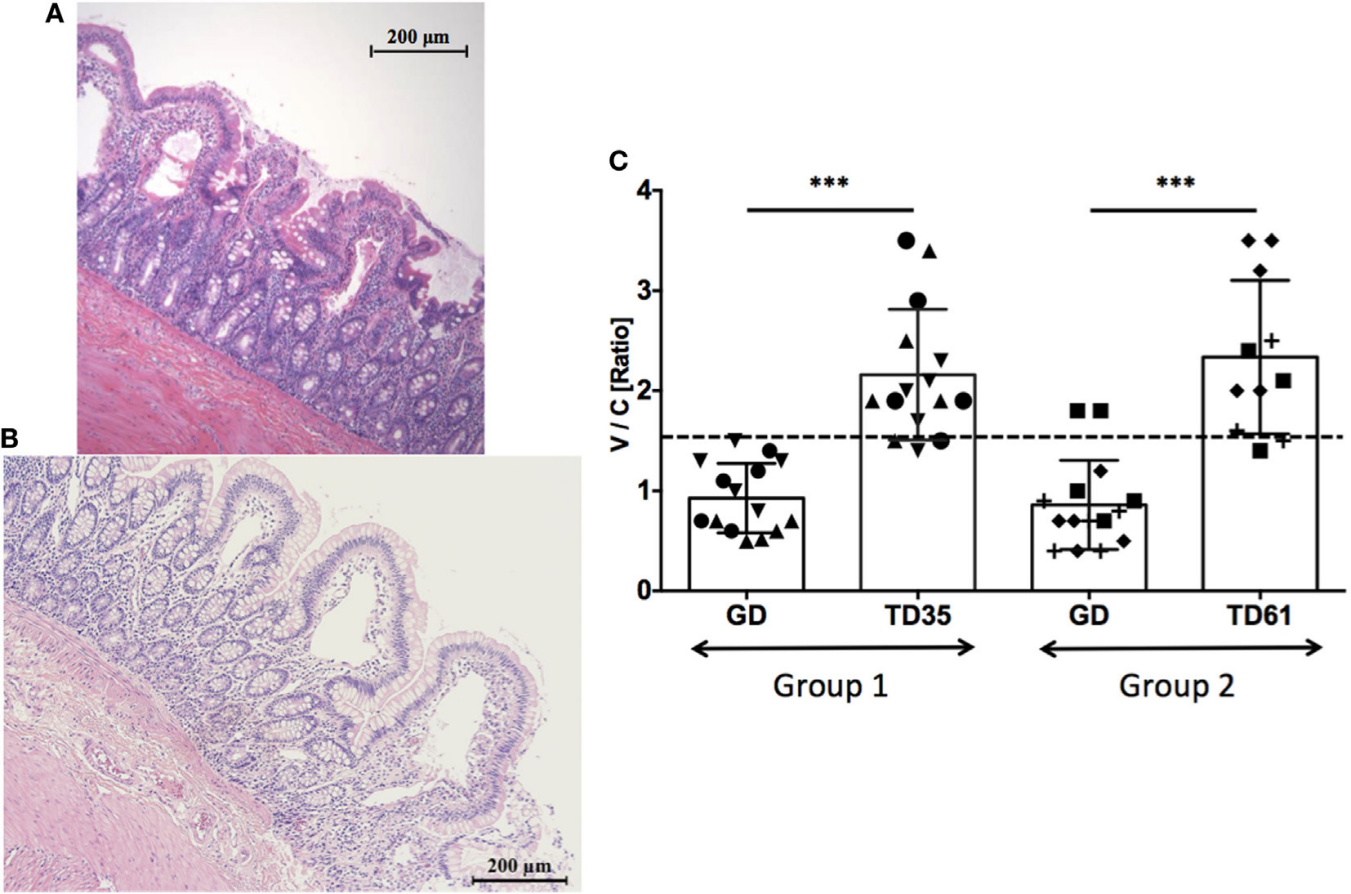
Jejunum tissue architecture (H&E staining) upon anti-IL-15 treatment. The macaque LI75 with moderate enteropathy (Type 2) characterized by fusion, shortening, and blunting of some of the villi at the time of first biopsy **(A)**. Impact of anti-IL-15 treatment after 61 days **(B)** is shown when second set of biopsies was collected. Morphometric data combined from both groups of macaques revealed restoration of small intestinal architecture, i.e., villous heights vs. crypt depths ratios (V/C) upon 35 days of treatment in group 1 (TD35) and 61 days of treatment in group 2 (TD61) macaques; ***P < 0.001 **(C)**. Group 1: five measurements per each animal LD17 (▲), LE73 (▼), LF68 (●), group 2: LE99 (◆), LD09 (◼), LI75 (?), and time point, are shown. Healthy control, GD-fed macaque baseline (≥1.5) is indicated by dashed line.

To evaluate efficacy of anti-IL-15 treatment in gluten-sensitive, celiac-like macaques, the small intestinal tissue architecture was compared between biopsy samples representing the gluten-induced antibody relapse (gluten-containing diet = GD), remission (gluten-free diet = GFD), and anti-IL-15 treatment while on gluten-containing diet (Figures 1 and 2; Figure S1 in Supplementary Material). First biopsy was collected at time when gluten-containing diet was fed for at least 6 months, and AGA were significantly (*P* < 0.05) elevated above those of healthy controls. For the anti-IL-15 treatment phase, the six GSE macaques were divided into two groups of 3 based on severity of enteropathy; mild (group 1) and moderate (group 2).

Microscopic examination of H&E stained jejunum biopsy tissues collected from studied macaques prior, during, and after the anti-IL-15 treatment revealed enteropathy with subsequent amelioration upon anti-IL-15 treatment (Figures 2A–C). Morphometric evaluation of small intestinal tissue architecture, i.e., villous heights vs. crypt depth ratio was performed (Figure 2C) to assess the significance of individual morphological observations. Villous height/crypt depth ratio data corroborated that anti-IL-15 treatment benefited (*P* < 0.001) both groups of celiac macaques, as all treated animals exhibited increased heights of small intestinal villi, upon treatment to an extent comparable with healthy, age-matched controls (Figure 2C) (20).

### Anti-IL-15 Treatment Reduces Numbers of Small Intestinal Intraepithelial CD3+ T Lymphocytes

**Figure 3:**
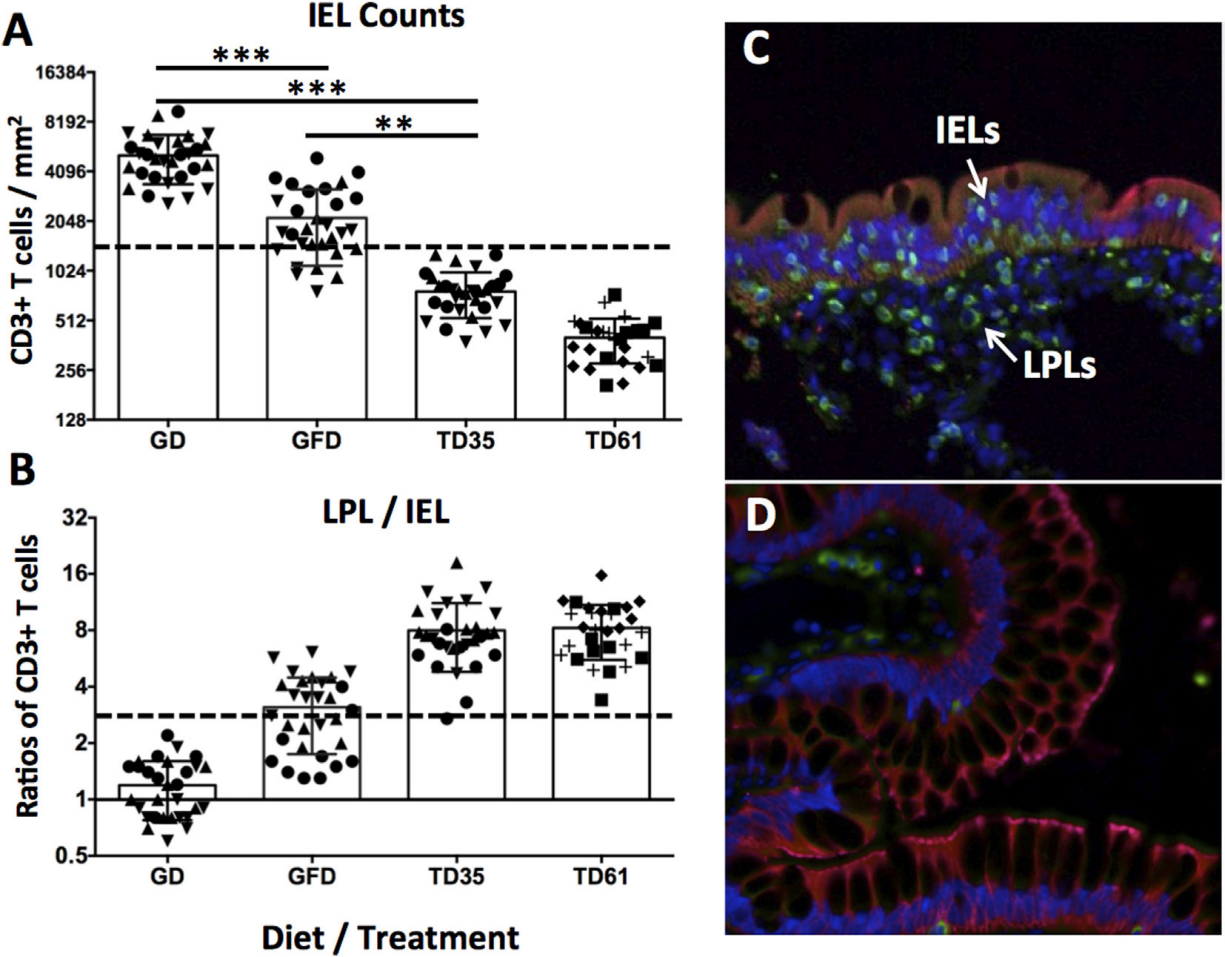
Counts of small intestinal intraepithelial lymphocytes (IELs) and LPL/IEL ratios. Observed IEL counts **(A)** and LPL/IEL ratios **(B)** correspond to combined values of three gluten-sensitive enteropathy (GSE) macaques at each time point (10 × mm^2^ counts per animal and time point). Macaques LD17 (▲), LE73 (▼), and LF68 (●), LE99 (◆), LD09 (◼), and LI75 (?), are indicated **(A,B)**. Dashed lines indicate healthy control baselines. Time point reflect 6 months of GD diet **(C)**, 3 months of GFD diet, 35 and 61 days of anti-IL-15 treatment in Group 1 and Group 2, i.e., TD35 and TD61 **(D)**, respectively. The epithelial cells (cytokeratin 1+) are in red, the CD3+ T cells are in green, and To-Pro+ (DNA) cell nuclei are in blue **(C,D)**. The IELs within the GSE-affected epithelium and LPLs beneath the epithelium are indicated by arrows (**C**). ***P < 0.001, **P < 0.01.

To further evaluate efficacy of anti-IL-15 antibody treatment in macaques with GSE, the counts of small intestinal IELs and lamina propria lymphocytes vs. IELs ratios were compared between biopsy samples taken at time points representing the GD diet (6 months), GFD (3 months), and anti-IL-15 treatment (days 35 and 61) while on GD (Figure 3).

Remarkably, following anti-IL-15 treatment, intraepithelial lymphocyte counts were improved (i.e., reduced) not only relative to gluten-containing diet (*P* < 0.001) but also relative to gluten-free diet levels: despite being fed gluten-containing diet, anti-IL-15 treatment, measured at post-treatment; day 35 (TD35) in Group 1 macaques and at TD61 in Group 2 macaques, resulted in a greater (*P* < 0.01) decrease of IELs than that associated with 3 months of gluten-free diet (Figure 3A). Ratios between small intestinal lamina propria lymphocytes and IELs inversely mirrored IELs counts (Figure 3B). The CD3+ lamina propria lymphocytes and IELs were compartmentalized within the small intestinal lamina propria and epithelium, respectively, as illustrated (Figure 3C). The two biopsies taken from gluten-sensitive macaque, LI75, during GD and treatment periods are shown to illustrate the anti-IL-15 treatment efficacy (Figures 3C,D). Reduced numbers of intraepithelial and lamina propria lymphocytes at treatment day 61 (Figure 3D) show that anti-IL-15 antibody treatment was effective.

### Anti-IL-15 Treatment Reduces Numbers of Small Intestinal Lamina Propria-Derived CD8+IFN-γ+ and CD4+IFN-γ+ T cells

**Figure 4:**
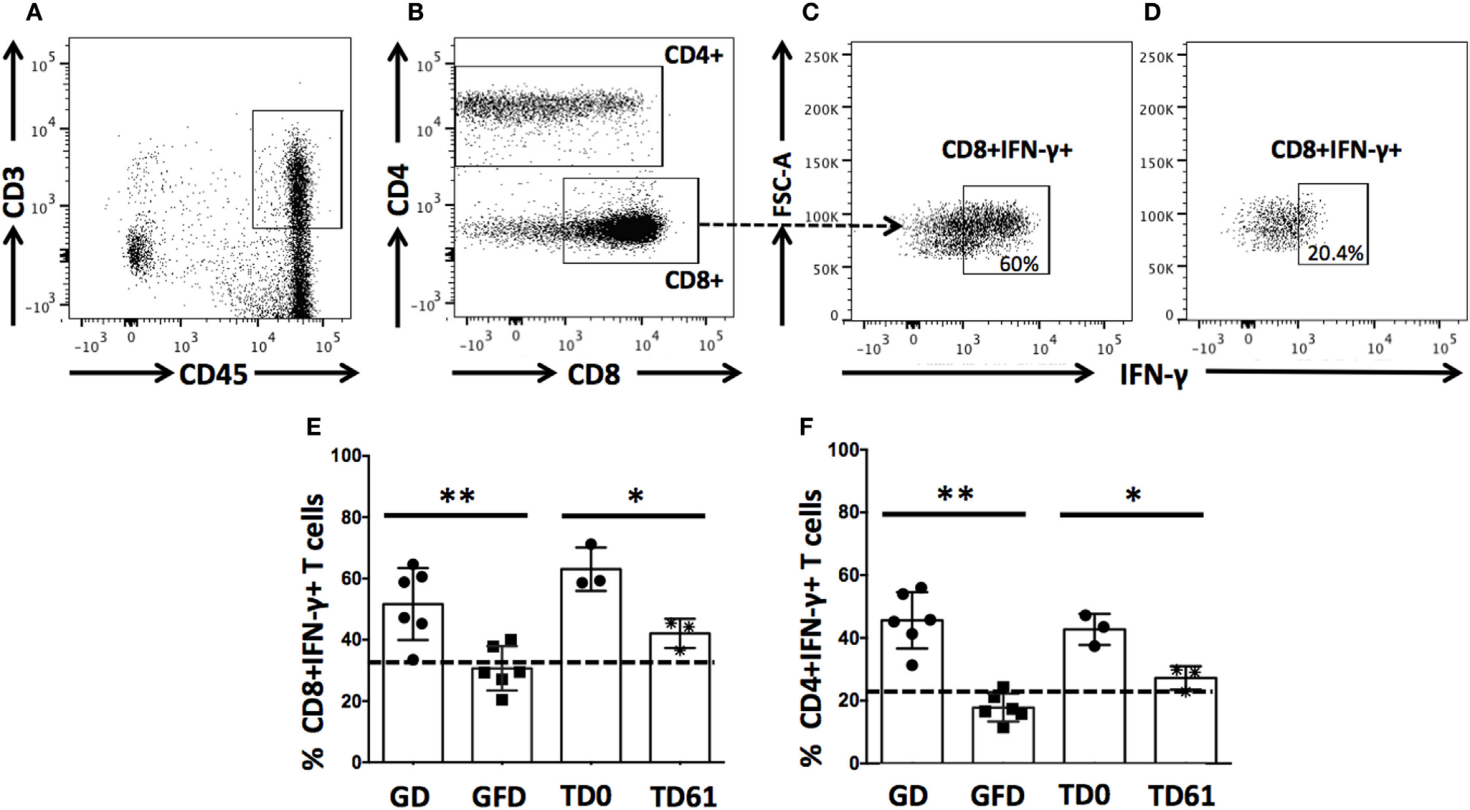
Anti-IL-15 treatment reduces IFN-γ production by intestinal CD8+ and CD4+ T cells in gluten-sensitive enteropathy (GSE) macaques. Small intestinal lamina propria-derived CD3+CD45+ T cells **(A)** were defined as parent population. From the parent cells, CD4−CD8+ and CD4+ (including both CD4+CD8− and CD4+CD8+ subsets) T cells **(B)** were selected and evaluated for presence of IFN-γ. Impacts of GD **(C)**, GFD **(D)**, and anti-IL-15 **(E,F)** treatments are shown: a steady decrease (**P < 0.01) of CD8+IFN-γ+ and CD4+IFN-γ+ T cells was noted within 90 days of GFD in all six GSE macaques **(E,F)**. A similar but less robust (*P < 0.05) effect was seen in anti-IL-15-treated Group 2 macaques by TD61 when small intestinal biopsy was taken again **(E)**. Baseline levels corresponding to healthy control macaque are indicated by dashed lines **(E,F)**.

Due to the effector role CD8+ and CD4+ T cells play in pathogenesis of celiac disease, the impact of anti-IL-15 treatment on small intestinal CD3+CD4−CD8+CD45+ and CD3+CD4+CD8−/+CD45+ T cells secreting IFN-γ was evaluated (Figures 4A–F). Consistent with past reports (12, 22), 90 days of gluten-free diet reduced counts (*P* < 0.01) of both CD8+IFN-γ+ and CD4+IFN-γ+ T cells in all six macaques with GSE, back to baseline levels and not different from healthy controls. In Group 1 macaques prior to anti-IL-15 treatment, counts were within the baseline and not different from healthy controls (not shown). In Group 2 macaques with severe enteropathy, counts of CD8+IFN-γ+ and CD4+IFN-γ+ T cells were elevated also prior to treatment. Anti-IL-15 treatment lead to reduced counts of intestinal CD8+IFN-γ+ and CD4+IFN-γ+ T cells (*P* < 0.05) in Group 2 macaques although not entirely to levels associated with healthy controls or gluten-free diet (Figures 4E,F).

### Anti-IL-15 Treatment Reduces Numbers of Peripheral NK but Not Effector Memory T Cells

**Figure 5:**
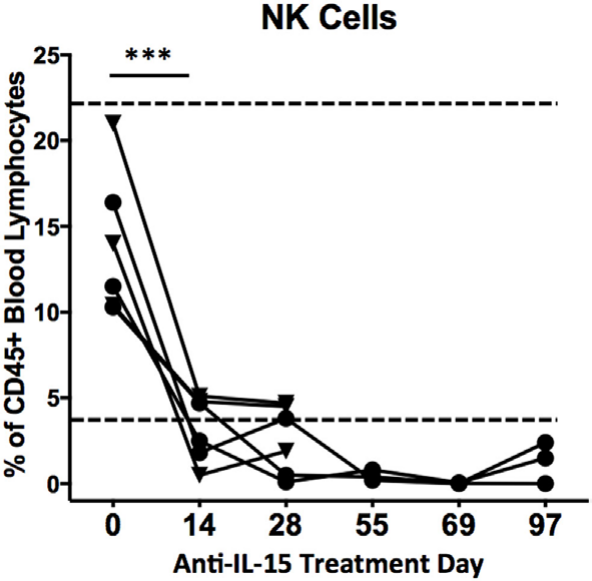
Proportions (% of CD45+ lymphocytes) of CD3−HLADR−CD8a+NKG2D+ cells in peripheral blood following anti-IL-15 treatment. A decrease in NK cell counts, ***P < 0.001, took place within 14 days of initiation and continued for the duration of treatment. Proportions of NK cells from CD45+ lymphocytes are shown for both group 1 (▼) and group 2 (●) macaques up to treatment day 28 while group 2 animals are shown up to TD 97, i.e., 7 days post final anti-IL-15 dosing. A healthy control macaque range is indicated by dashed lines.

**Figure 6:**
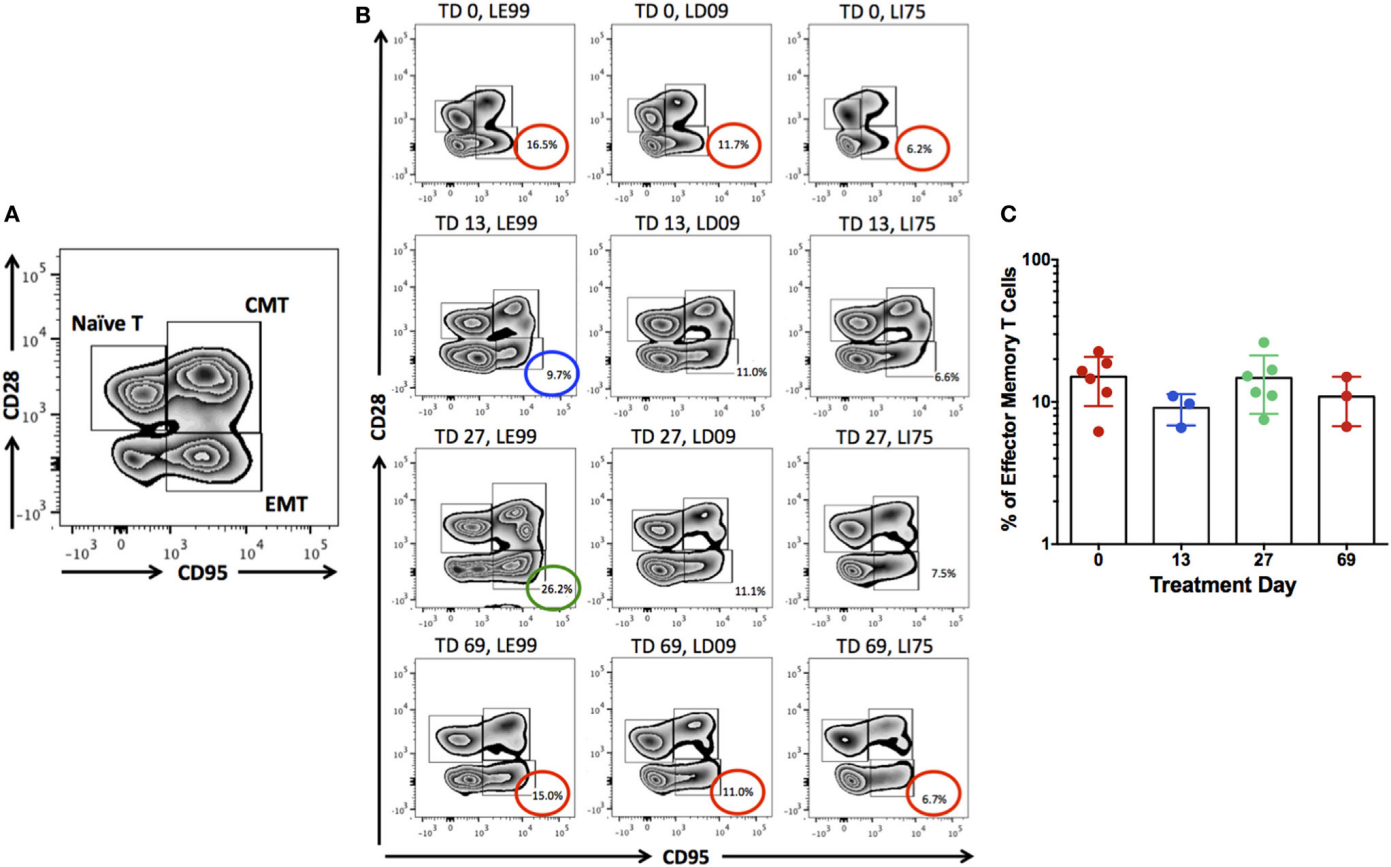
The populations of peripheral naïve (CD3+CD28+CD95−), central memory (CD3+CD28+CD95+), and effector memory (CD3+CD28−CD95+) T cells were identified **(A)**. Proportions of effector memory T cells (% of CD3+ lymphocytes) were examined for the potential depleting effects by anti-IL-15 antibody at treatment day (TD) 0, 13, 27, and 69 **(B,C)**. Although some macaques (LE99) showed lowered counts of effector memory T cells by TD 13 (blue circle), followed by expansion at TD 27 (green circle) and normalization (red circle) at TD 69 **(B)**, no significant effects (*P* > 0.05) were noted at the group level **(C)**.

Previous studies have indicated that inhibition of IL-15 in healthy macaques leads to changes in the peripheral NK and possibly also effector memory T cells numbers (16). Thus, we sought to determine if anti-IL-15 treatment exerted predicted, NK and effector memory T cell-depleting effects in GSE macaques (Figures 5 and 6). The proportions of peripheral blood NK cells (CD3−CD45+HLADR−CD8a+NKG2D+) within the population of blood lymphocytes were monitored during the course of anti-IL-15 treatment, along with other major lymphoid populations, including T cells (CD3+), effector memory T (CD3+CD28−CD95+), monocyte/B-cells (CD3−HLADR+), T helper (CD3+CD4+), and cytotoxic T (CD3+CD8+) cells (Figures 5 and 6A; Figure S2 in Supplementary Material). NK cells were further subdivided by expression of CD16 and CD56 antigens (Figure S3 in Supplementary Material). In agreement with expected effects and as early as 14 days post anti-IL-15 treatment initiation, reduced counts (*P* < 0.001) of NK cells, beneath the pretreatment levels, were detected in all six macaques (Figure 5). These reduced counts of peripheral NK cells persisted for the duration of anti-IL-15 treatment. No significant reductions were observed in case of peripheral T cells including CD3+CD28−CD95+ effector memory T, monocyte/B, T helper, cytotoxic T cells, serum chemistry or hematology values (Figure 6; Table 2; Figure S2 in Supplementary Material) despite that decreased counts of effector memory T cells were observed by treatment day 13 in some animals the example of which is LE99 (Figure 6B). Overall, however, such decrease was not significant (*P* > 0.05), and normalized in subsequent time points while treatment was ongoing (Figures 6B,C).

**Table 2:** Selected values of clinical chemistry and hematology.

Value	Unit	Base-range	GD	GFD	Anti-IL-15 treatment		
					
					1 month	2 months	3 months
Albumin	g/dl	3.0–5.9	4 ± 0.3	3.9 ± 0.3	3.9 ± 0.2	4.3 ± 0.4	4.0 ± 0.3
Globulin	g/dl	1.9–3.9	2.6 ± 0.3	2.7 ± 0.2	2.5 ± 0.2	2.8 ± 0.2	2.9 ± 0.3
Sodium	mmol/l	144–160	146 ± 4.1	145 ± 1.5	147 ± 2.0	147 ± 0.6	145 ± 1.2
Chloride	mmol/l	106–117	106 ± 3.8	109 ± 1.4	108 ± 3.0	106 ± 2.1	108 ± 2.6
Potassium	mmol/l	3.3–6.4	3.3 ± 0.2	3.6 ± 0.1	3.8 ± 0.4	3.9 ± 0.3	3.8 ± 0.3
Glucose	mg/dl	48–119	82 ± 21.2	73 ± 12.7	69 ± 13.1	82 ± 2.7	74 ± 5.6
BUN	mg/dl	13–27	18.2 ± 0.8	19.7 ± 3.3	22.2 ± 3.6	22.7 ± 3.2	21.3 ± 2.1
Creatinine	mg/dl	0.4–1.4	0.5 ± 0.04	0.6 ± 0.2	0.6 ± 0.1	0.6 ± 0	0.6 ± 0.1
Hemoglobin	g/dl	10.1–15.9	12 ± 0.7	12.1 ± 0.6	11.6 ± 1.3	13.5 ± 0.5	12.6 ± 0.2
Hematocrit	%	34.8–55.2	37.8 ± 1.8	38.1 ± 1.9	37.8 ± 3.4	43 ± 1.4	40.4 ± 0.8
WBC	10(9)/l	6.6–15.5	10.5 ± 2.1	7.1 ± 3.2	8.6 ± 3.2	10.7 ± 4.6	10.3 ± 5.9
RBC	10(12)/l	4.1–7.8	5.6 ± 0.4	5.5 ± 0.3	5.4 ± 0.5	6.0 ± 0.1	5.7 ± 0.3
PLT	10(9)/l	193–676	455 ± 73	489 ± 55	496 ± 33	651 ± 31	529 ± 80
AST	U/l	25–120	42.3 ± 4.8	53.3 ± 17.6	32.7 ± 4.2	50 ± 14.1	36.7 ± 5.0
ALT	U/l	20–126	29 ± 5.1	55.5 ± 25.6	32.3 ± 7.8	33 ± 1.7	28.7 ± 1.2

*GD, gluten-containing diet; GFD, gluten-free diet; BUN, blood urea nitrogen; WBC, white blood cells; RBC, red blood cells; PLT, platelet; AST, aspartase aminotransferase; ALT, alanine aminotransferase*.

### Anti-IL-15 Treatment Reduces Anti-Gliadin Plasma Antibodies

**Figure 7:**
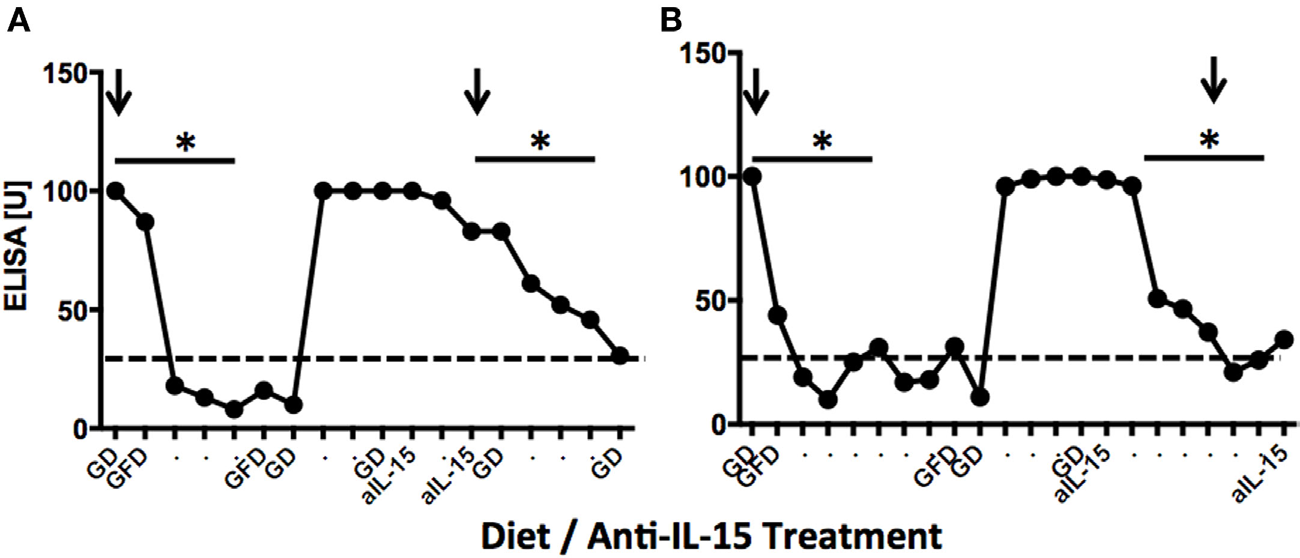
Anti-gliadin antibodies (AGA) responses in two representative gluten-sensitive enteropathy (GSE) macaques. Dynamics of anti-gliadin IgG antibodies in LE73 **(A)** and LI75 **(B)** macaques reflecting experimental diets and anti-IL-15 treatments of Group 1 and Group 2 animals, respectively. Distances between time points correspond to 2-week intervals. Negative base line (healthy control macaque) levels are indicated by dashed lines. Arrows indicate times when jejunum biopsies were collected. LE73 **(A)** had mild GSE at the time of GD biopsy; therefore, anti-IL-15 treatment was administered for 28 days only. LI75 **(B)** had pronounced GSE, thus, anti-IL-15 treatment was in this animal administered for 90 days. In both animals, anti-IL-15 treatment resulted in significant (*P < 0.05) decline of plasma AGAs to negative control levels.

To examine the effects of anti-IL-15 treatment on anti-gliadin and TG2 antibodies in macaques with GSE while on gluten-containing diet, the plasma levels of above antibodies were compared between samples collected during gluten-induced behind anti-gliadin antibodies (AGA) relapse, remission, and anti-IL-15 treatment while on gluten-containing diet (Figures 1 and 7). First anti-gliadin antibody relapse time point reflected the time when gluten-containing diet was fed for at least 6 months and antibodies were elevated (*P* < 0.05) above those of healthy controls (Figure 7).

Gluten-free diet regimen led to remission during which AGA antibodies decreased (*P* < 0.05) to baseline in all six GSE macaques (Figures 7A,B). With exception of LD17, reintroduction of dietary gluten resulted in rapid expansion of anti-gliadin antibody but much slower and less robust expansion of TG2 autoantibodies (Figure S4 in Supplementary Material). Following anti-IL-15 treatment period, AGA antibodies showed second decline (Figure 7). Lowered anti-gliadin antibody persisted for 2 months of anti-IL-15 treatment in Group 2 animals despite that macaques were fed gluten-containing diet. Within third month of treatment, both AGA and TG2 antibodies started to rise again in two out of three (Group 2) animals (Figure S4 in Supplementary Material). In Group 1 macaques, close-to-baseline levels of both antibodies persisted beyond treatment for at least 2 months. This was in contrast with period following gluten-free diet when anti-gliadin antibody started to rise within 3 weeks after replacement of gluten-free with gluten-containing diet.

## Discussion

To enable experimental and preclinical studies toward evaluation of new therapies, over the past 10 years, our group has developed the rhesus macaque model of GSE (18–20, 22). Consistent with human celiac disease, GSE in macaques is characterized by a wide range of severity, ranging from the subclinical to severe form that includes decreased absorption of nutrients, decreased xenobiotic metabolism, cancer predisposition, diarrhea, dermatitis, decreased diversity of gut microbiome, presence of TG2 autoantibodies, and MHC II-mediated genetic predisposition (13, 18, 19). Long-term gluten-containing diet in this model induces antibody and T cell responses that can be modulated by reduction of dietary gluten intake and leads to restoration of small intestinal architecture (12, 15). Consequently, progress of mucosal integrity repair is one of the key factors that govern recovery from celiac symptoms. In agreement with human spectrum of disease, in macaques with GSE, the antibody responses to gliadin and TG2, as well as enteropathy, range widely. The relapse of AGA in comparison with relapse of TG2 antibodies in celiac macaques is faster, as it occurs within several weeks after the reintroduction of dietary gluten. TG2 antibody relapse requires longer time, i.e., at least several months. We hypothesize that this is due to fundamentally different mechanisms of diet-induced anti-gliadin vs. autoimmune TG2 antibody production. As small intestinal pathology varies in different individuals between mild submucosal inflammation and complete villous atrophy consistent with celiac disease, the length of gluten-free diet period required for restoration of small intestinal epithelial integrity in macaques with GSE is not uniform. Usually, at least 2 months are required to restore the mucosal architecture in macaques with mild GSE (12, 20). In cases of severe enteropathy characterized by flattening villous atrophy, thorough mucosal restoration might not be accomplished even after 6 months of gluten-free diet. Thus, alternative and gluten-free diet supplementary therapeutic approaches capable of accelerating small intestinal mucosa recovery are critically needed.

Intestinal immune cells, notably CD3 + IELs, exert pathological changes in small intestinal mucosa of both celiac patients and macaques with GSE (12, 20, 25, 26). Numbers of these cells and the extent of associated tissue damage can be used as metrics of disease progression or improvement, following the administration of novel experimental drugs or therapies (12, 27). IEL phenotypes include not only CD3+ T cells but also CD3− NK and NKT cells (27–29). Increased expression of IFN-γ and invariant TCRα chain of human NKT cells (Vα24-Jα18) was demonstrated by FACS of duodenal IELs from celiac patients (28). Four types of human intestinal CD3− IELs were defined based on co-expression of CD56 and CD127 antigens (29). It was shown that plasticity and diversity of these IEL populations diminish with progression of enteropathy (29). It was recommended that >30% increase in IEL counts is significant in the context of celiac disease clinical trials (30). In our study, IEL counts surpassed Tampere’s celiac threshold in enteropathic macaques while on gluten-containing diet. Interestingly, despite continued intake of dietary gluten, anti-IL-15 treatment resulted in macaques with GSE in faster and greater decrease of IELs (*P* < 0.001) than that associated with 3 months of gluten-free diet (Figure 3A). Such a rapid and thorough decline of small intestinal IELs to baseline levels was mirrored with restoration of V/C ratios, decline of intestinal CD8+IFN-γ+ and CD4+IFN-γ+ T cells, suggesting the beneficial impact of anti-IL-15 treatment (Figures 2–4). The histopathological examination of small intestinal mucosa from GSE macaques corroborated these effects: after completing 2 months of anti-IL-15 treatment, intestinal architecture became morphologically close to normal healthy mucosa (Figure 2). Interestingly, anti-IL-15 treatment did not significantly alter counts of peripheral effector memory T cells although in some macaques (LE99), these cells decreased transiently and returned to normal during the treatment period (Figure 6). Such result is to an extent consistent with study that focused on a different anti-IL-15 antibody (M111) where depletion of effector memory cells in treated macaques was followed by the onset of massive proliferation (16).

Within the third month of treatment, anti-gliadin and TG2 antibodies started to rise again above baseline levels in two out of three (Group 2) macaques. This was not due to lack of antibody exposure, as the serum concentrations of the anti-IL15 antibody in both groups of macaques were greater than 75 μg/mL starting from treatment day 7 onward (Figure S5 in Supplementary Material), indicating elevated levels of antibody in the treatment phase of the study. It would be of interest to evaluate in future studies, if antibody remission in affected macaques could be improved and extended, by combining anti-IL-15 treatment with another supplementary treatment and/or gluten-free diet. Since in juvenile macaques with less developed forms of GSE, clinical scores of diarrhea and stool consistency are less reliable predictors of disease remission/relapse than immunological or histopathological measurements, we did not use clinical diarrhea as metric in this study.

An anti-IL-15 antibody, which like 04H04 inhibits IL-15 receptor signaling, has been clinically tested in celiac disease patients (NCT02633020, NCT02637141). However, no outcomes have been reported from these studies, and hence, the only available data on anti-IL-15 efficacy in celiac-like disease is in preclinical animal models. A model that recapitulates selected features of celiac disease, by utilizing transgenic mice overexpressing human IL-15 in the intestinal epithelium has been employed to investigate the role of IL-15 in disease pathogenesis (31). This model exhibits accumulation of IELs in the intestine and villous atrophy (10). Two different anti-IL-15 antibodies have been shown to reverse the accumulation of IELs in this model (17, 32). With the presence of anti-gliadin and anti-TG2 antibodies linked with genetic predisposition and enteropathy, the GSE macaque model used in this study provides fully translatable model to human celiac disease. Our study is the first to describe that anti-IL-15 therapy is beneficial in preventing severe GSE including compromised epithelial integrity, inflammation and loss of small intestinal tissue architecture in a clinically relevant rhesus macaque model of celiac disease.

The CD3−CD45+HLADR−CD8a+NKG2D+ phenotype was used to delineate parent population of NK cells by flow cytometric analysis, with subsequent further division into CD56+ and/or CD16+ subsets. From the seven markers used to define rhesus peripheral NK cells, the NKG2D antigen was particularly critical, as it not only represents primary activation receptor of NK/NKT cells but it also plays role in pathogenesis of autoimmune diseases including CD (4, 33, 34). Rhesus/human cross-reactive CD16 and CD56 antibodies were employed previously to distinguish NK cells in SIV-infected macaques (35–37). It remains to be seen whether the administration of the human anti-IL-15 antibody used in this study would have similar NK depletion effects in humans. For now, we can conclude that the effects of human anti-IL-15 therapy in GSE macaques are consistent with those produced by other anti-IL-15 antibodies, specifically reduced numbers of peripheral NK cells, IELs and enteropathy improvement (17, 38). In clinical trials conducted thus far, administration of anti-IL-15 antibody (AMG 714) did not result in lowered peripheral NK cell counts, potentially due to IL-15-dispensable mechanism regulating the maintenance of human peripheral NK cells (38). Given that 04H04 and AMG 714 both block IL-15 binding to the IL-15 signaling receptor complex, we expect no modulation of NK cell numbers by 04H04 in humans, and this would be monitored closely in clinical development. In summary, our study is the first to demonstrate that human anti-IL-15 antibody therapy in rhesus macaques with GSE attenuates gluten-induced small intestinal mucosal injury (improved V/C ratio), mucosal inflammation (reduced IEL counts) and plasma AGAs, all clinically relevant outcomes for human disease. Thus, anti-IL-15 antibody has potential for the treatment of celiac disease and other inflammatory diseases in which IL-15 is involved.

## Ethics Statement

This study was performed using samples collected from non-human primates. All procedures were approved by the TNPRC Institutional Animal Care and Use Committee, Animal Welfare Assurance A-4499-01, and were performed in accordance with the *Guide for the Care and Use of Laboratory Animals, National Research Council, 2011*. The TNPRC maintains an AAALAC-I accredited animal care and use program.

## Author Contributions

KS wrote the manuscript, coordinated the overall work, participated in work related to multi-color flow cytometry, analyzed the data, and interpreted the findings. JPD provided veterinary care, executed surgical procedures, and helped with coordination of dietary regimens. DXL was responsible for histopathological evaluation of intestinal biopsies. XA directed immunohistochemistry and confocal microscopy work. NR was responsible for directing the intestinal lymphocyte isolation procedures. JB helped with preparation and coordination of animal studies. DJL, AF, and AGD were responsible for anti-IL-15 material generation, study design, and interpretation. AWC was responsible for the study’s overall management and interpretation.

## Conflict of Interest Statement

DJL, AF, AGD, and AWC are employees of Teva Pharmaceuticals. All other authors declare no competing interests.

## Supplementary Material

Figure S1 HE-stained image of normal, healthy control jejunum tissue architecture from juvenile rhesus macaque **(A)** in comparison with age-matched jejunum tissues from gluten-sensitive enteropathy (GSE) macaques with mild/Type 1 **(B)** while on GD. Type 1 GSE in macaques is characterized by intraepithelial lymphocytosis **(C)** but not villous atrophy.

Figure S2 No effects of anti-IL-15 treatment beyond ranges corresponding to age-matched controls (pink-shaded areas) were observed in peripheral blood lymphocyte populations from group 1 **(A)**, and group 2 **(B)** macaques: the CD45+ lymphocytes were characterized as CD3+HLADR− (T cells), CD3−HLADR+ (B/monocytes), CD3+CD4+ (T helper), and CD3+CD8+ (cytotoxic T) cells.

Figure S3 Proportions (% of CD45+ lymphocytes) of CD3−HLADR−CD8a+NKG2D+ cells in peripheral blood following anti-IL-15 treatment. A decrease in NK cell counts (*P* = 0.0002) took place within 14 days of treatment (TD 14) and continued for the duration of treatment.

Figure S4 Anti-gliadin antibodies and anti-intestinal tissue transglutaminase plasma antibodies in six gluten-sensitive enteropathy macaques selected for the study. Distances between the time points correspond to 2 weeks. Healthy control base-lines are indicated by dashed lines.

Figure S5 The individual pharmacokinetic profiles of serum anti-IL-15 antibody in groups 1 and 2 macaques. Arrows indicate the timing of anti-IL-15 treatments.
