# Supplementary material for: Beneficial Effects of Human Anti-Interleukin-15 Antibody in Gluten-Sensitive Rhesus Macaques with Celiac Disease
Source: Front Immunol. 2018 Jul 11;9:1603. doi: 10.3389/fimmu.2018.01603 (PMC6050360; doi:10.3389/fimmu.2018.01603)
Supplement: Figure S1 — HE-stained image of normal, healthy control jejunum tissue architecture from juvenile rhesus macaque (A) in comparison with age-matched jejunum tissues from gluten-sensitive enteropathy (GSE) macaques with mild/Type 1 (B) while on GD. Type 1 GSE in macaques is characterized by intraepithelial lymphocytosis (C) but not villous atrophy. [file image_1.PDF]

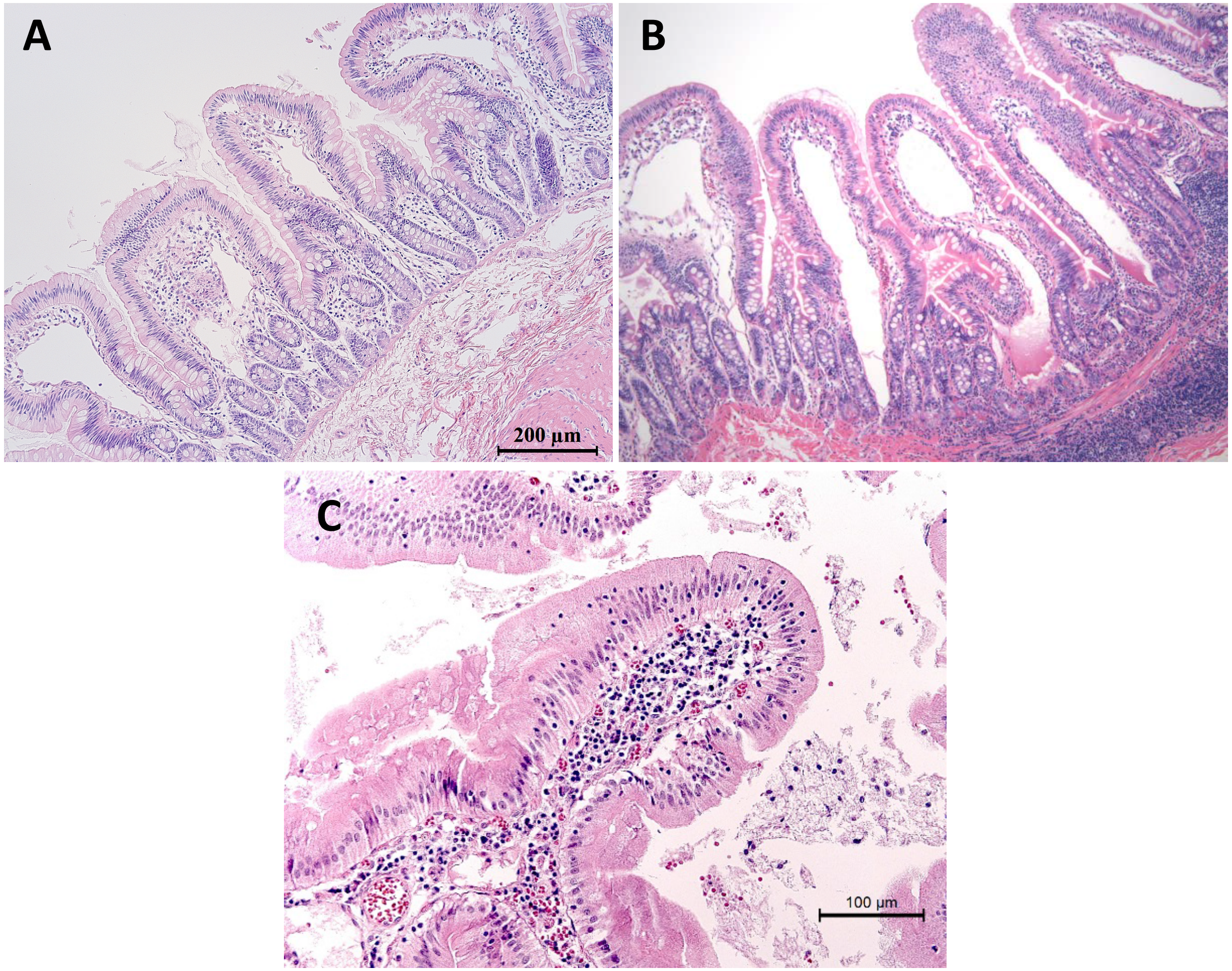

Supplemental Figure S1. HE-stained image of normal, healthy control jejunum tissue architecture from juvenile rhesus macaque (**A**) in comparison with age-matched jejunum tissues from GSE macaques with mild/Type 1 (**B**) while on GD. Type 1 GSE in macaques is characterized by intraepithelial lymphocytosis (**C**) but not villous atrophy.
