# Supplementary material for: Beneficial Effects of Human Anti-Interleukin-15 Antibody in Gluten-Sensitive Rhesus Macaques with Celiac Disease
Source: Front Immunol. 2018 Jul 11;9:1603. doi: 10.3389/fimmu.2018.01603 (PMC6050360; doi:10.3389/fimmu.2018.01603)
Supplement: Figure S2 — No effects of anti-IL-15 treatment beyond ranges corresponding to age-matched controls (pink-shaded areas) were observed in peripheral blood lymphocyte populations from group 1 (A), and group 2 (B) macaques: the CD45+ lymphocytes were characterized as CD3+HLADR− (T cells), CD3−HLADR+ (B/monocytes), CD3+CD4+ (T helper), and CD3+CD8+ (cytotoxic T) cells. [file image_2.PDF]

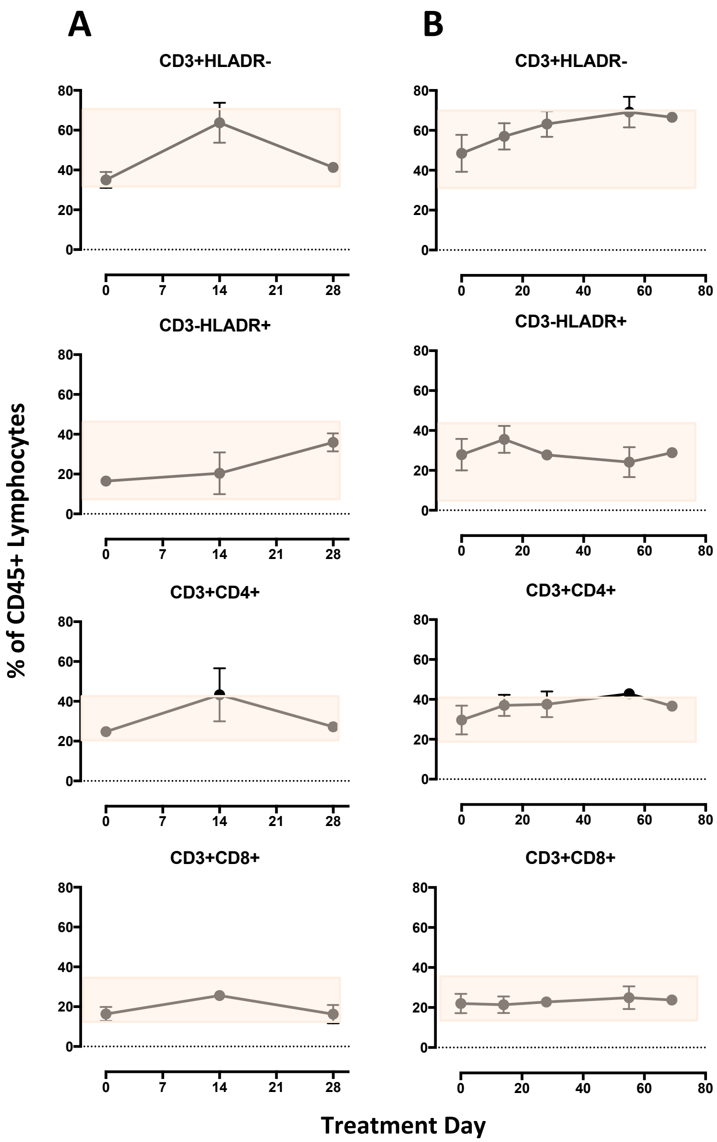

Supplemental Figure S2. No effects of anti-IL-15 treatment beyond ranges corresponding to age-matched controls (pink-shaded areas) were observed in peripheral blood lymphocyte populations from group 1 (A), and group 2 (B) macaques: The CD45+ lymphocytes were characterized as CD3+HLADR- (T cells), CD3-HLADR+ (B / monocytes), CD3+CD4+ (T helper) and CD3+CD8+ (cytotoxic T) cells.
