# Supplementary material for: Beneficial Effects of Human Anti-Interleukin-15 Antibody in Gluten-Sensitive Rhesus Macaques with Celiac Disease
Source: Front Immunol. 2018 Jul 11;9:1603. doi: 10.3389/fimmu.2018.01603 (PMC6050360; doi:10.3389/fimmu.2018.01603)
Supplement: Figure S3 — Proportions (% of CD45+ lymphocytes) of CD3−HLADR−CD8a+NKG2D+ cells in peripheral blood following anti-IL-15 treatment. A decrease in NK cell counts (P = 0.0002) took place within 14 days of treatment (TD 14) and continued for the duration of treatment. [file image_3.PDF]

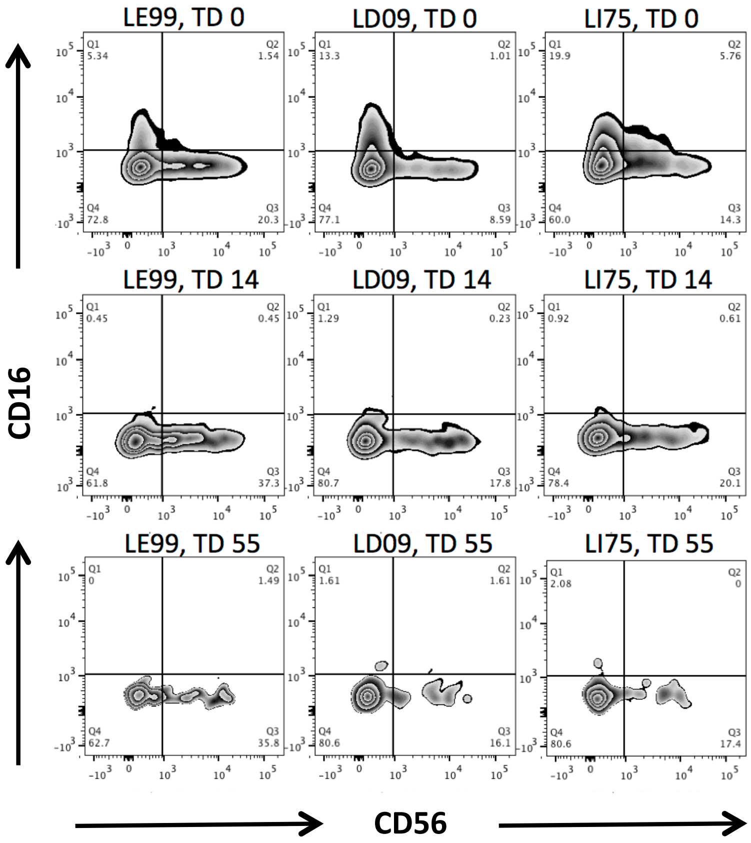

Supplemental Figure S3. Proportions (% of CD45+ lymphocytes) of CD3-HLADR-CD8a+NKG2D+ cells in peripheral blood following the anti-IL-15 treatment. A decrease in NK cell counts (P=0.0002) took place within 14 days of treatment (TD 14) and continued for the duration of treatment.
