# Supplementary material for: Beneficial Effects of Human Anti-Interleukin-15 Antibody in Gluten-Sensitive Rhesus Macaques with Celiac Disease
Source: Front Immunol. 2018 Jul 11;9:1603. doi: 10.3389/fimmu.2018.01603 (PMC6050360; doi:10.3389/fimmu.2018.01603)
Supplement: Figure S4 — Anti-gliadin antibodies and anti-intestinal tissue transglutaminase plasma antibodies in six gluten-sensitive enteropathy macaques selected for the study. Distances between the time points correspond to 2 weeks. Healthy control base-lines are indicated by dashed lines. [file image_4.PDF]

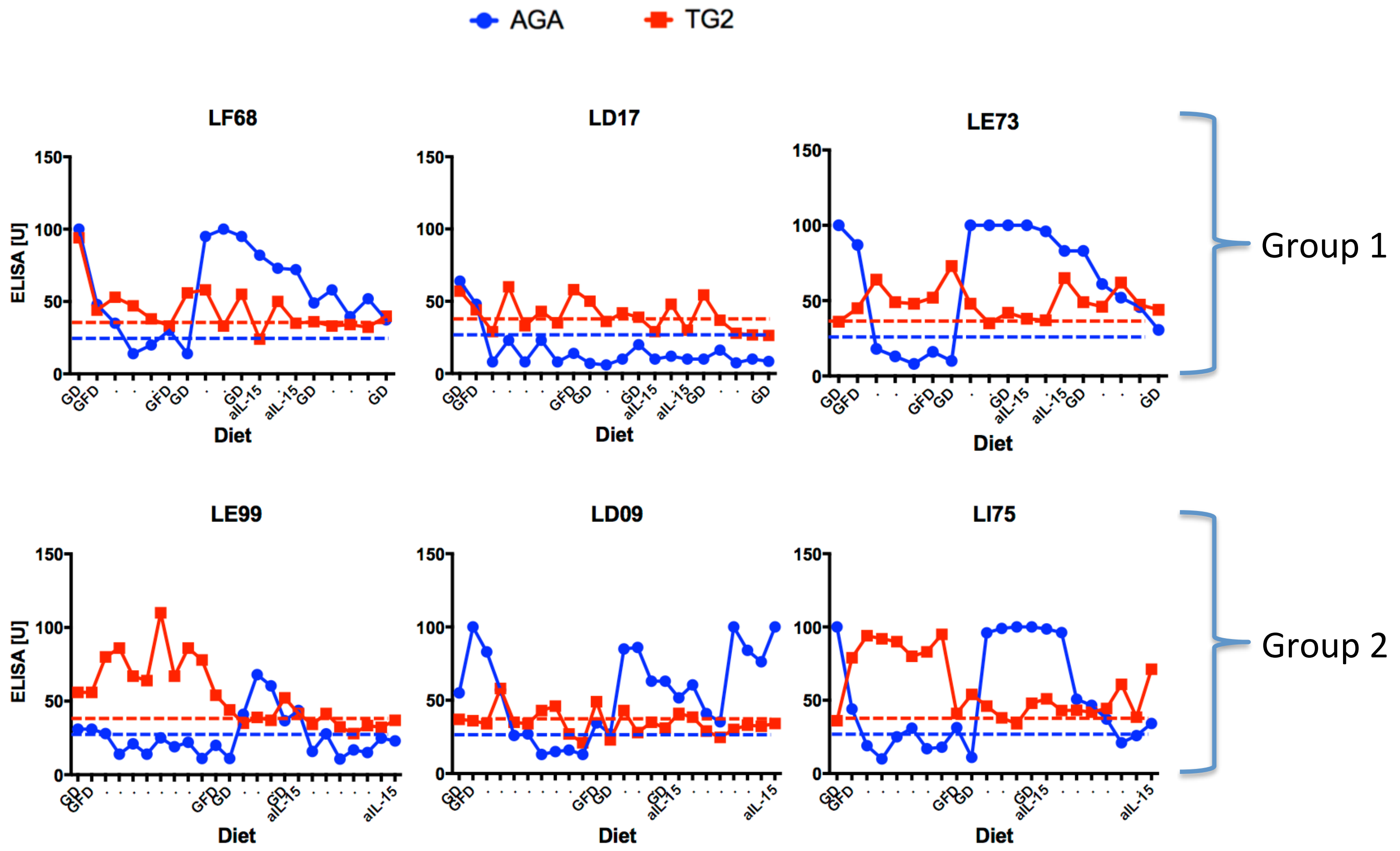

Supplemental Figure S4. Anti-gliadin antibodies (AGA) and anti-intestinal tissue transglutaminase (TG2) plasma antibodies in 6 GSE macaques selected for the study. Distances between the time points correspond to 2 weeks. Healthy control base-lines are indicated by dashed lines.
