# Supplementary material for: Beneficial Effects of Human Anti-Interleukin-15 Antibody in Gluten-Sensitive Rhesus Macaques with Celiac Disease
Source: Front Immunol. 2018 Jul 11;9:1603. doi: 10.3389/fimmu.2018.01603 (PMC6050360; doi:10.3389/fimmu.2018.01603)
Supplement: Figure S5 — The individual pharmacokinetic profiles of serum anti-IL-15 antibody in groups 1 and 2 macaques. Arrows indicate the timing of anti-IL-15 treatments. [file image_5.PDF]

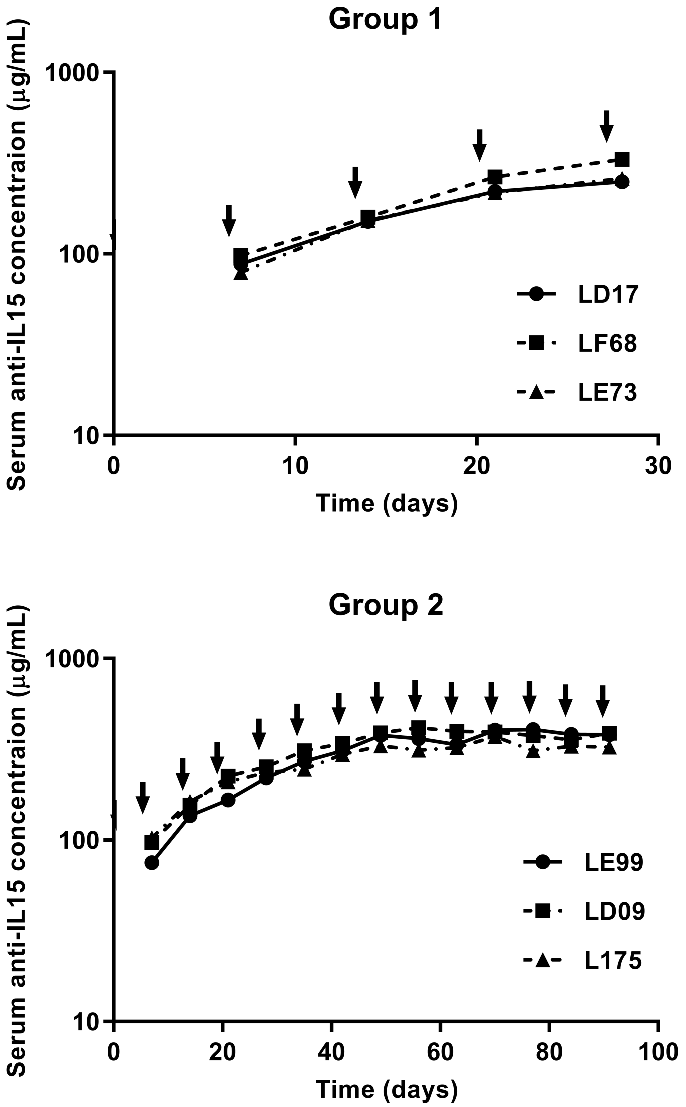

Supplemental Figure S5. The individual pharmacokinetic profiles of serum anti-IL-15 antibodies in groups 1 and 2 macaques. Arrows indicate the timing of anti-IL-15 treatments.
